# American Medical Association

**Published:** 1878-07

**Authors:** 


### American Medical Association.

The opening general session of the meeting of the American Medical Association was held June 4th at St. James Hall, in this city. The main floor of the hall was filled by eleven o'clock.

The Association was called to order at 11.10 A. M., by the President, Dr. T. G. Richardson, of New Orleans Among those who occupied the stage were the President, the Vice-Presidents, and the Secretaries. After the session had been called to order, prayer was offered by the Rev. L. Van Bokkelen, D. D., rector of Trinity Church.

Profs. Gross, Davis, Toner, and Bowditch, ex-Presidents of the Association were invited to seats upon the stage. Then came the address of welcome by Dr. Thomas F. Rochester, of Buffalo, chairman of the Committee of Arrangements, which was appropriate and well received by the association.

The Secretary then presented the report of the Committee of Arrangements on the credentials of members. He read the list of 330 names from the Registry-book, and the report was adopted.

The Secretary announced that he had received charges against various medical societies, and some applicants for admission to membership, and the same were, on motion, referred to the Judicial Council.

The Committee of Arrangements presented a report recommending the reception of all the members of the Erie County Medical Society not delegates, and the following persons, by invitation:

George W. Stoner, M. D, United States Marine Hospital Service.
F. Lange, M. D., Kiel, Germany.
Edward Hutchinson, M. D. of Utica, N. Y.

They also presented for election as permanent members, the following:

George I. Northrup, M. D., of Marquette, Mich.
Stanford E. Chaille, M. D., of New Orleans, La.
L. G. Thacker, M. D., of Defiance, O.

The Secretary next read the following cablegram from Paris:

President American Medical Association—May your meeting be harmonious and contribute more than ever to the advancement of medicine. Truly sorry I cannot be with you. MARION SIMS.

The Chairman of the Committee of Arrangements made some announcements for the future proceedings and the programme of entertainments, after which Prof. James P. White introduced the President, T. G. Richardson, M. D. of Louisiana, who delivered his annual address.

At the conclusion of the address Prof. White said that it was idle for the Association to meet year after year and hear such propositions as had been presented by the President, without adopting some plan which would mature the same.

Prof. Gross moved that the thanks of the society be tendered to Dr. Richardson for his "excellent, very able, highly elaborated, scholarly and finished address" and expressed the opinion that the enthusiasm thrown into it had doubtless been infused into all present, and he hoped it would bear fruit of a high order in the future. The motion was carried.

On motion of Prof. White, a committee consisting of the President and his four immediate predecessors, was appointed to take into consideration the suggestions and recommendations set forth in the address.

Dr. Wm. Brodie, of Detroit, then read his report as the representative to the Canadian Medical Association.

The report was referred to the Committee on Publication.

Dr. L. A. Sayre, of New York, made a verbal report of his visit as a delegate to the British Medical Association. The report was brief and practical, and highly satisfactory.

The report on the action of the American Delegation before the International Medical Congress of Geneva by Dr. E. Seguin, of New York, was then read by the Secretary. This was also referred to the Committee on Publication.

After some announcements had been made, the Association adjourned to meet in sections at 3 P. M.

*Section One*, Practical Medicine, Materia Medica, and Physiology convened at No. 354 Washington street, second story, at 3 o'clock in the afternoon. Dr. A. L. Loomis, chairman, presided, and in the absence of the regular secretary, Dr. G. H. Etheridge, of Illinois, Dr. J. Shoemaker, of Pennsylvania, was elected to fill the position. Dr. Shoemaker then proceeded to read an abstract of a paper on "Ring-worm in Public Institutions."

The next paper read was on "Pulmonary Tuberculosis," by by Dr. E. N. Davis, of Chicago. It was then discussed by Dr. N. S. Davis, of Chicago, and referred to the Committee on Publications."

Dr. Glasgow presented a specimen of fibrinous bronchitis, and remarks were made by Dr. Loomis, of New York, in which he stated he had seen six cases of this rare disease, and found that the influence of climate controls it. Dr. Rich moved to have Dr. Glasgow report a full history of the case at the next meeting. The paper was referred.

*Section Two*—Obstetrics and Diseases of Women and Children convened at No. 354 Washington street, third floor, and was largely attended. Dr. E. W. Jenks, of Detroit, presided, and Dr. H. O. Marcy, of Cambridge, Mass., officiated as Secretary. A paper on "Ovotomy" was read by Dr. Theophilus Parvin, of Indiana. It was discussed by Dr. Miller, of Chicago, who endorsed Dr. Parvin's theory, and by Dr. Reaves, of Ohio, Dr. Jenks, Dr. Dunster, of

Michigan, and others. Attention was called to operations performed by Drs. White and Miner, of this city.

*Section Three*—Surgery and Anatomy, met at St. James Hall in the afternoon, and was called to order by the Chairman, Dr. Henry H. Smith of Philadelphia, at three o'clock. The Secretary, Dr. E. T. Easley of Little Rock, Arkansas, performed the usual duties of that office.

In accordance with the rules, a sub-committee was appointed to examine papers read.

Dr. Howe of this city presented a patient and gave a verbal account of a case of plastic surgery upon the eye, accompanied with photographs.

Dr. C. C. F. Gay, of Buffalo, read a paper on the "Excision of the Diaphysis of the Tibia."

Dr. S. H. Weeks, of Portland, Maine, read a paper on "septicæmia following Resection of Bones."

Dr. Waterhouse asked the differance between pyæmia and septicæmia.

Dr. Weeks replied that by pyæmia he understood the reception into the blood of pus or some of its elements. In septicæmia there is decomposing blood. There are other points of difference, as there are metastatic abscesses in pyæmia, but not usually in septicæmia.

Dr. Keller had found that opium did not produce sleep except in doses dangerously large. Quinine could be given in large doses.

Dr. Woodward referred to the difference between septicæmia and pyæmia, and wished to know if the gentleman's statements were made on his own observation. He had seen blood poisoning without the existence of pus, but had never seen pus in the blood, or found anybody who had.

Then came a paper from Dr. Henry A. Martin, of Boston, on the subject of "Tracheotomy without Tubes." The next paper was read by Dr. John D. Carpenter, of Pottsville, Pa., on the "Identity of Hospital Gangrene with Diphtheria."

The Chairman read a letter from Dr. D. M. Clay, of Shreveport, La , regretting his inability to be present.

Dr. Wyckoff, of this city, was introduced, and read Dr. Clay's paper on "Peri-typhlitic Abscess."

This completed the regular programme, but two volunteer papers were read—the first by Dr. Post, on "Plastic Surgery;" and the second by Dr. E. M. Moore, of Rochester, on "Prevention of Septicæmia."

*Section Four*—For the consideration of Medical Jurisprudence, Chemistry and Psychology, had no papers, and did not organize. The Chairman, Dr. Walter Kempster, of Oshkosh, Wis., the Secretary, Dr. E. A. Hildreth, of Wheeling, W. Va., and a few others, met at the Fireman's Benevolent Association room, and adjourned.

*Section Five*—Met in the lecture room of Washington Street Baptist Church, at three o'clock, for the consideration of State Medicine and Public Hygiene, Dr. J. D. Cabell of the University of Virginia presided, and as soon as the Section was called to order, Dr. Henry I. Bowditch, of Boston, read an interesting paper upon "Studies of Epidemic of the Diphtheria which prevailed at Ferrisburg (adjacent to Vergennes) Vermont, 1877." The locality and course of the epidemic were traced out with great care and presented ciscumstantially. He presented the salient points of the epidemic and the paper was an exceedingly interesting one.

At the conclusion of his paper, Dr. Bowditch moved that his paper be referred to a committee of experts. Pending the vote on this motion, the paper was discussed by Dr. Jacobi and Dr. Bell of New York, Dr. Hollister of Chicago, Dr. Noyes of Detroit, Dr. Seguin of New York.

Dr. Toner moved as an amendment to the motion of Dr. Bowditch, that the paper be referred to a committee for publication. Dr. Jones of Toledo, in seconding the motion, took occasion to speak of contagion and infection, their causes, etc.

After further remarks by Dr. Jacobi, and Dr. Knight, and Dr. Isham of Connecticut, the vote was taken on the amendment of Dr. Toner, and it was carried.

Dr. E. Seguin of New York, next read an essay on the "Intervention of Physicians in Education." He spoke of the injury to the health of children by badly-ventilated school-rooms, and advocated the interference of doctors in our educational systems to remedy these evils. The paper was discussed by Dr. Bell, of New York; Dr. Jones, of Toledo; Dr. F. H. Hamilton, of New York; Dr. Noyes, of Detroit; and Dr. Storer, of Rhode Island. On motion of the latter, Dr. Hamilton was requested to present the subject before the Association, together with an outline of Dr. Seguin's views.

THE SECOND GENERAL SESSION.—The President, Dr. T. G. Richardson, of New Orleans, called the meeting to order. On the stage were the vice-presidents, secretaries, and several of the most distinguished physicians. Dr. Clark and Dr. Trenholme, members of the Canadian Medical Association, and Dr. Botsford, were also invited to seats there.

The list of delegates registered subsequent to the general session of Tuesday was read by the Secretary.

Letters of regret received from Dr. B. A. Vaughn, of Columbus, Miss., and Dr. Robert Battey, of Rome, Ga., were read and directed to be entered in the minutes.

The Judicial Council then submitted their report.

The Judicial Council would respectfully present the following report:

The charge against Dr. J. M. Keller, presented by Dr. G. W. Lawrence, were dismissed as unworthy of any consideration,

The charges against the Society of Grand Rapids, Mich., by Dr. P. J. Dwyer, are incomplete and unaccompanied by witnesses and testimony whereby the Judicial Council might be able to act.

The Arkansas petition referring to the State Medical Society, were dismissed without action. The charges against the Michigan State Society by Dr. W. W. Jones will be reported upon by Dr. N. S. Davis, Chairman of the Judicial Council.

The charges against W. T. Barr, M. D. of Abingdon, Va., presented by Dr. Ulrich of Chester, Pa., were dismissed by the Council.

S. N. Benham, M. D., Secretary of Judicial Council, in reference to the communication from the State Medical Society of Arkansas notifying the Association that the Hot Springs and Garland County Medical Society was not recognized by the State Medical Society and protesting against the reception of any members of that Society, it is decided that under the by-laws of this Association, said Hot Springs and Garland County Society loses its recognition with this Association from the date of its severance from the State Society.

S. N. Benham, M. D.,
Secretary Judicial Council.

The above was received and adopted.

Dr. N. S. Davis, Chairman of the Judicial Council, read the following additional report:

Report of the Judicial Council concerning the charges against the Michigan State Medical Society, referred by the Association at the annual meeting in June, 1877.

The charge in this case was an alleged violation of the code of ethics of the Michigan State Medical Society, in electing as a delegate to this Association Dr. E. L. Dunster, of the University of Michigan, knowing him to be engaged in aiding and abetting the graduation of students devoted to an exclusive dogma in medicine.

After a most careful examination of the code of ethics as it has appeared in the transactions of this Association from year to year, the Judicial Council fail to find any section or paragraph in it, that refers, even remotely, to the practice that constitutes the foundation of the charge under consideration, that any member of the medical profession proper, should ever engage in teaching, examining and certifying to the qualification of students, knowing that such teaching and examinations, were to aid said student in obtaining a diploma directly admitting them into a fraternity of irregular practitioners, was evidently not contemplated by the framers of our Code of Ethics, and hence they inserted no clause or section bearing upon that subject. The only provision in the code referring to those engaged in an attempt to practice medicine in accordance with "exclusive dogmas," is in the section regulating consultations at the bed-side of the sick. If the Judicial Council of this National organization should assume that the section of the code just referred to, indicated "the spirit" of those who framed and adopted it, and on that assumption, apply it to matters and practises entirely foreign to those mentioned in the section itself, it would not only violate all the accepted principles of judicial construction, but would establish a precedent in latitudinous construction of the ethical code more dangerous to the best interests of the profession than all the evils sought to be remedied in the case under consideration.

It is true that this Association has adopted at different times two resolutions having reference to the subject involved in the charge against the Michigan State Medical Society, which still stand as expressions of opinion unrepealed.

But these resolutions constitute no part of the code of ethics; neither is obedience to them enjoined by the Constitution and By-laws on State and local medical societies as a condition of representation in this Association.

Therefore, while deprecating the practice of aiding or abetting in any way the teaching and graduation of students known to be supporters of singular and exclusive dogmas in medicine, as beneath the dignity of rightminded teachers of an honorable and liberal profession, your Judicial Council can find no clause in either the constitution, by-laws or Code of Ethics as they now exist, under which the charge against the Michigan State Medical Society can be entertained and adjudicated.

An animated discussion followed the reading of the report, and was participated in by Drs. J. M. Toner, N. S. Davis, S. C. Busey, J. J. Woodward, Wm. Brodie and Thomas Menees. The first named moved to refer the report back to the Judicial Council, and the same was seconded by Dr. Busey. Dr. Menees offered an amendment to the effect that the report be referred to the Council with instructions to draft a law governing the defect complained of and embracing a penalty for the punishment of the violation of the law. Before any action could be taken, the hour for the next order of business arrived.

Prof. Henry H Smith, Chairman of the section of Surgery and Anatomy, then delivered his address, taking for his subject: "Certain points on the Pathology of the Bones, especially Tubercle." After alluding to the opinion of former years that the skeleton was intended to support the body, he mentioned that recently a new function had been assigned it, the bones being now regarded by many as a "focus for the origin of the white and red corpuscles of the blood and through which serious diseased matter was introduced into the general circulation."

After showing its connection with septicæmia and blood poison, he discussed the original progress of tubercles in the bones, and their influence on Pott's and hip disease, both of which he thought were of tubercular origin. It was referred to the Publication Committee.

Dr. Frank H. Hamilton, of New York, by request, next presented the essential features of the able paper prepared by Dr. E.

Seguin, of New York, on "The Intervention of Physicians in Education," read by the author before the Section on State Medicine and Public Hygiene.

On motion of Dr. Toner the paper of Dr. Seguin was referred back to the section for publication.

When Dr. Hamilton had concluded the reading of extracts from the paper he said that it was hoped that the paper would be well discussed. Accordingly he spoke at some length upon the subject matter, dealing principally with the hygienic affairs in New York, and emphatically endorsed the paper.

Owing to the lateness of the hour further discussion, the President announced, was unavoidably prevented. Dr. Hamilton, therefore offered the following resolution, which was unanimously adopted :

*Resolved*, That in the opinion of this Association medical men ought to have a voice in the construction and location of public school buildings; in the question as to the age at which children should be admitted, the hours of study, and the general management of these institutions; and to this end it is believed to be necessary that one or more intelligent physicians should be placed upon Boards of Education, Boards of Trustees, and upon other similar boards having the control of public education and schools.

Moses Gunn, M. D., Vice-President, took the chair, and Dr. E. W. Jenks, of Detroit, Mich., Chairman of the Section on Obstetrics and Diseases of Women and Children, delivered an address on "The Causes of Sudden Death of Puerperal Women." The paper was an able and exhaustive one and was referred to the Section on Obstetrics for publication.

Action upon the report on the revision of the By-Laws was made the special order of business for Thursday.

Dr. J. R. Bronson, of Massachusetts, offered the following preamble and resolutions :

*Whereas*, By the report of the Judicial Council submitted this day, we are informed that the ethical code of this Association is imperfect in that it does not recognize by its letter a conceded violation of the spirit of our profession in its relation to irregular medicine. Therefore,

*Resolved*, That said Council be instructed to submit to this Association at their meeting for its consideration an amendment to the Code covering this omission.

The above was on motion referred to all the members of the Judicial Council as a Committee.

Dr. Foster Pratt, of Kalamazoo, Michigan, offered a resolution in relation to the legal status of the insane, which was referred to the section on Medical Jurisprudence.

The Association then adjourned to meet in sections.

### THE SECTION MEETINGS.

*In Section One*—The first paper read was on yesterday's programme by Dr. George M. Beard, of New York, entitled: "Practical Points in the electrical treatment of Impotence and Spermatorrhea."

Dr. Beard's paper was discussed at some length by Dr. Caldwell of Indiana, Palmer of Michigan, Bronson of Massachusetts, Shoemaker of Philadelphia, Woodbury of Philadelphia. It was referred to a sub-committee, consisting of Drs. Palmer, Hibbard and Bronson.

Dr. J. J. Caldwell, of Baltimore, then proceeded to read a paper on "The Neuroses of the Pneumogastric and Sympathetic."

A paper on "The Metric System in Medicine," by Dr. Edward Wigglesworth, of Massachusetts, was read. The system, he stated, has been recommended for use throughout the United States by the State Medical Societies of both New York and Pennsylvania. The section then adjourned.

*Section Two*—In this section, the first paper read was by Dr. T. A. Reamy, of Ohio, on "Hourglass Contraction of the Uterus," &c. Dr. Reamy's paper was discussed by Dr. Miller of Chicago, Dr. Dunster of Michigan, Dr. Parvin of Indiana, Dr. Baker of Milesboro, Dr. Bontecue of Troy.

Dr. H. Storer of Rhode Island, then read a paper on "The frequently Gynecological Origin of Inherited forms of Strumous Disease, and the consequent indications of treatment." The paper was referred to a committee.

*Section Three.*—Dr. Moses Gunn, of Chicago, was called to the chair temporarily, and Dr. Easley, of Little Rock, Arkansas, was at his post as Secretary.

Dr. H. H. Smith, of Philadelphia, presented his demonsrrations on certain points in the "Pathology of the Bones." At the end of the demonstrations, Dr. Smith resumed charge of the proceedings of the Section.

On motion it was decided that a debate of one half an hour take place on the subject presented by Dr. Smith, each speaker being limited to five minutes. The discussion was participated in by Dr. Gouley, and Dr. Sayre of New York.

Prof. Julius F. Miner, of Buffalo, then read a paper on "The Extirpation of the Thyroid Gland," illustrating it with specimens of tumors and of glands which had been entirely removed. The subject was discussed by Drs. Miner and Sayre. Following came a paper from Dr. B. A. Watson, of Jersey City, on "Disease Germs, their Origin, Nature and Relation to Wounds.

On motion of Dr. Post, of New York, all papers read in the section were ordered to be referred to a special committee. Dr. Hodgen, of St. Louis, and Dr. Hutchinson, of Brooklyn, were appointed such committee.

A paper was then read by Dr. Frederick Hyde, of Cortland, N. Y., on "The Process of Repairs in Wounds, with and without Antiseptic treatment."

Dr. Robert Burns, of Philadelphia, followed with a treatise on "Conservative Surgery in Compound Fractures."

Dr. Robert Battey, Rome, Ga., sent a letter regretting his inability to be present, but forwarded the paper which he was announced to read. It was on "The Permeability of the entire Alimentary Canal by Enemata, with some of its Surgical Applications." It was referred to the sub-committee.

The final paper of the afternoon, was read by Dr. Post, of New York, on "The Excision of Phalanges of the Fingers and Toes."

*Section Four.*—Held its first meeting in the rooms of the Firemen's Benevolent Association. Dr. Walter Kempster, Chairman presided, and Dr. W. M. Compton, of Mississippi, was chosen Secretary *pro tem.* Dr. Kempster read an interesting and lenghty paper on the "General Paresis of the Insane."

*Section Five.*—The subject of croup and diphtheria as contagious diseases was discussed by Prof. Jacobi, of New York; Dr. Wilcox, of Hartford; Dr. Abbott, of Hamburgh; Dr. R. J. O'Sullivan, of Mew York; Dr. Reeves, of Wheeling, and others.

Dr. Bell, of New York, then offered the following resolution, which was adopted.

*Resolved,* That Dr. E. Sequin's paper on the Intervention of Physicians in Education be recommended for publication in the transactions, and that Drs. F. H. Hamilton, E. Seguin, R. J. O'Sullivan, of New Yark; Dr. D. B. Lincoln, of Boston; Dr. W. H. Van Bibbin, of Baltimore, be appointed a committee to report to the Association at its next meeting upon the practical suggestions of the said paper.

Dr. J. S. Billings, U. S. A., of Washington, exhibited the plans of the John Hopkins Hospital, to be erected at Baltimore, showing improved plans of ventilation and drainage.

On motion, several papers which had been submitted were referred to a committee, consisting of the President and Secretary of the Section, with power.

The Third General Session—Opened at twenty minutes to 10 o'clock with President Richardson in the Chair, and the other officers in their places.

The list of additional delegates was read by Secretary Atkinson.

The Secretary also read a letter of regret from Dr. R. O. Howard, of Montreal.

A protest to the registration of a New York State delegate was read and referred to the Judicial Committee.

The Judicial Council reported that the charges against the Iowa State Medical Society, and Scott County Medical Society, presented by Dr. P. J. Farnsworth, are dismissed, because the subject had been already adjudicated by the Council at their Detroit meeting in 1874 They also reported that in the matter of the Washtenaw County Medical Society, it was decided that the Ann Arbor Society be entitled to two delegates to the present meeting. The report went over for one year.

Dr. Alfred Post, of New York, moved that the first two on the list from the Ann Arbor Medical Society be received as delegates, the others remaining as members by invitation. Carried.

Dr. A. N. Bell, of Garden City, offered the following resolution, which was laid over until next year:

*Resolved*, That Section Four, on Medical Jurisprudence and Psychology, and Section Five, on State Medicine and Public Hygiene, be consolidated into one section as Section Four.

Dr. N. S. Davis, of the Judicial Council, presented the following report proposing an amendment in addition to the Code of Ethics, and action on the same was deferred according to the rules for one year:

In obedience to the instructions of this Association, the Judicial Council, acting in the capacity of a Committee, have unanimously instructed me to report to your Honorable Body the following amendment and addition to paragraph 1, article 1, of the second division of the Code of Ethics, under the general heading, "Of the duties of physicians to each other, and to the profession at large," and the special heading, "Duties for the Support of Professional Characters." The same, when finally adopted, to be added at the end, and constitute a part of said paragraph 1, of article 1. The proposed addition is in these words: "And hence it is considered derogatory to the interests of the public, and the honor of the profession, for any physician or teacher to aid, in any way, the medical teaching or graduation of persons knowing them to be supporters and intended practitioners of some irregular and exclusive system of medicine."

Dr. J. M. Toner, of Washington, D. C., Chairman of the Committee on Necrology, presented his annual report. It was a very voluminous document, containing sketches of seventy-five physicians who had died during the the past year, and owing to its great length was not read but referred at once to the Committee on Publication.

The Secretary reported tnat the Committee on Catalogue of National Library was not prepared to make a report, and asked to to be continued. The request was granted.

Dr. A. L. Loomis of New York, Chairman of the section on Practical Medicine, Materia Medica and Physiology, was then introduced and delivered an able, exhaustive and interesting address. He noticed some of the important advances made in Practical Medicine, Physiology and Materia Medica, and also discussed at length the climatic treatment of pulmonary phthisis.

Dr. James P. White, of this city, offered the following resolution:

*Resolved,* That a committee of five be appointed to confer with General Myer, upon the subject of making observations as to the existence of ozone in various localities, and take such other steps and measures in the matter as may be necessary for the success of the object.

In seconding the above, Dr. Davis, of Chicago, briefly stated what had already been done to attain the object by the profession since 1874. After a discussion by Drs. Busey, White, Bell, Bronson, Leonard, Loomis and Davis, the resolution was adopted.

Dr. Toner, of the Committee on Nominations, announced that they were ready to report. Accordingly Dr. Hupp, of West Virginia, read the following, which was, on motion of Dr. White, of this city, accepted and adopted unanimously:

### REPORT OF THE COMMITTEE ON NOMINATIONS.

After due consideration the Committee on Nominations respectfully report that they have nominated the following gentlemen for the various offices named, to wit:

President—Theophilus Parvin, M. D., of Indiana.

Vice-Presidents—A. J. Fuller, M. D., of Maine; W. F. Westmoreland, M. D., of Georgia; John Morris, M. D., of Maryland; John H. Murphy, M. D., of Minnesota. Treasurer—Richard Dunglison, M. D., of Pennsylvania. Librarian—Wm. Lee, M. D., of District of Columbia. Committee on Library—John Eliot, M. D., of District of Columbia. Next place of meeting—Atlanta, Georgia. Time of meeting—The first Tuesday in May, 1879. Assistant Secretary—Scott Todd, M. D., of Atlanta, Georgia. Committee of Arrangements--J. P. Logan, Chairman; H. V. M. Miller, G. G. Crawford, H. L. Wilson, J. F. Alexander, J. M. Johnson, Charles Pinckney, V. H. Taliaferro, J. T. Johnson, of Atlanta, Ga. Committee on Prize Essays—Robert Battey, of Rome, Ga.; J. G. Westmoreland, of Atlanta. Ga.; Wm. A. Love, of Atlanta, Ga.; Robert Kidley, of Atlanta, Ga.; Henry F. Campbell, of Augusta, Ga.; J. H. Van Deman, of Chattanooga, Tenn. Committee on Publication—Dr. Wm. B. Atkinson, Chairman; T. M Drysdale, M. D., A. Fricke, M. D., S. D. Gross, M. D., C. Wister, M. D., R. J. Dunglison, M. D., of Pennsylvania, and Wm. Lee, M. D., of District of Columbia. The Committee also report the following nominations for Chairmen and Secretaries of Sections for 1879: I. Practice of Medicine, Materia Medica and Physiology—Dr. Thomas F. Rochester, of Buffalo, N. Y., Chairman; W. C. Glasgow, of St. Louis, Mo., Secretary. II. Obstetrics and Diseases of Women and Children—E. S. Lewis, of New Orleans, Chairman; J. R. Chadwick, of Boston, Mass., Secretary. III. Surgery and Anatomy—Moses Gunn, M. D., of Illinois, Chairman; Dr J. R. Weist, of Indiana, Secretay. IV. Medical Jurisprudence, Chemistry and Psychology

—Dr. Wm. M. Compton, of Mississippi, Chairman; L. M. Eastman, of Maryland, Secretary. V. State Medicine and Public Hygiene. —Dr. John S. Billings, of District of Columbia, Chairman; Dr. J. T. Reeve, of Wisconsin, Secretary. Committee on Necrology —J. M. Toner, M. D., of the District of Columbia, Chairman. Judicial Council—To fill a vacancy caused by death, John P. Gray, of Utica, N. Y.; in place of the seven whose terms expire at this meeting, D. A. Linthicum, of Arkansas; Foster Pratt, of Michigan; A. Woodward, of Connecticut; J. M. Toner, of District of Columbia; J. H. Van Deman, of Tennessee; S. M. Benham, of Pennsylvania; R. N. Todd, of Indiana.

After some announcements by the Committee on Invitations, Prof. Davis, of Chicago, presented the majority report of the Special Committee on the recommendations contained in the annual address of President Bowditch, delivered in Chicago last year. Dr. Bowditch then read a minority report.

The majority report was adopted unanimously.

R. J. Cabell, of the University cf Virginia, delivered a very able and exhaustive address which occupied the attention of the Association for nearly an hour upon State Medicine and Public Hygiene.

On motion the address of Dr. Cabell was referred to the Section on Hygiene.

Dr. Sayer then arose to a question of personal privilege and asked that the Secretary be requested to place him upon record as opposed to the resolution adopted at Detroit, which declared that a fracture of all long bones could not occur without shortening. The request was granted.

The Association then adjourned.

The several sections met in their respective quarters at three o'clock yesterday afternoon to hold their closing sessions.

Section One. In this Section Dr. Martin, of Boston, made some statements in reference to the use of the solid rubber bandage in cases of eczema and ulcers of the legs.

Dr. T. F. Rochester, of this city, proceeded to read a paper on "Separation of the Ileum, and Spontaneous Occlusion of the Divided Extremities," which was listened to with great interest. The subject was discussed by Dr. Palmer of Chicago, Dr. Loomis of New York and Dr. Martin of Boston. The paper was referred to the Committee on Publication.

Dr. C. N. Palmer's paper on the use of ergot in the cure of goitre was next read. Drs. Rochester, Blackwood, O'Hara, Hibbard and Hester took part in discussing this paper, which was referred.

The address of Dr. Loomis before the general session in the morning was then taken up and discussed at length. A resolution was adopted providing that Dr. Dennison of Colorado be requested to study the subject of the climatic treatment of pulmonary phthisis as he found it in Colorado and report at the next annual meeting. The section then adjourned *sine die.*

SECTION TWO. On reassembling yesterday afternoon a paper written by Dr. Levi F. Warner, of Boston, was read by Dr. Storer. The paper was discussed by Dr. Storer, Dr. Bartlett of Wisconsin, Dr. Marcy of Massachusetts, and was referred to the Committee on Publication.

Dr. John C. Irish's paper on Dr. Burnham's Surgical Treatment of Uterine Fibroids, was read by Dr. Dunster. The subject was briefly discussed by Drs. Storer, Smith and Dunster.

Dr. E. Cutter of Boston, spoke on the subject of the use of electrolysis for the same disease, and remarks were also made by Drs. Caldwell, Beard and Mussey.

Dr. George J. Engelman's paper on Battey's Operation for the Extirpation of the Ovaries was read by Dr. Dunster and briefly discussed.

The Section then adjourned *sine die.*

In SECTION THREE Dr. Hodgen of St. Louis presided in the absence of the regular Chairman. The first paper read was rather a lengthy one, by Dr. Frank H. Hamilton, of New York, his subject being, "Ex-section of the Meta-tarso-phalangeal Articulation in Valgus of the Great Toe."

On motion the regular order was suspended to allow Dr. Sayre to exhibit to the Section a child that had been treated and cured of Pott's disease by the application of the plaster jacket. The child had taken no medicine, and was free from any prominent deformity.

Dr. Gunn offered the following resolution:

*Whereas*, This Section having expressed an opinion upon the results of long bones: and

*Whereas*, In general convention a member has been asked and been accorded the privilege of recording his protesting vote; therefore

*Resolved*, That this Section re-affirms its opinion that shortening, in cases of fractures of the long bones, is the rule in practice, regardless of any of the means of treatment now in use.

The resolution was discussed by Drs. Kellar and Sayre in the negative, and by Dr. Frank H. Hamilton in the affirmative. The resolution was fully adopted, Dr. Sayre requesting that his protest against the opinion it expressed be placed on record.

Dr. John H. Packard of Philadelphia read a paper on "Fractures of the Wrist-joint," which was discussed by Dr. E. M. Moore of Rochester.

Dr. Theodore A. McGraw, of Michigan, read a paper on "The Pathology, Diagnosis and Treatment of Cancer." The next paper was by Henry O. Marcy, of Cambridge, Mass., on "A new use of Carbolized Ligatures as applied to Hernia."

After further discussion the section closed.

In SECTION FOUR the session was principally occupied in hearing read and discussing a paper on "Microscopic Examinations of the Nervous Centres," by Dr. Theodore Deecke, Special Pathologist of the Utica Insane Asylum. It was a paper of more than ordinary interest. The paper was referred, after discussion, and the Section adjourned.

In SECTION FIVE a letter from Dr. F. H. Hamilton of New York was read, declining to report at the next meeting of the Association on the subjects discussed in Dr. Seguin's paper, for the reason that he had not time to make such a report. A discussion then arose on the subject of the paper read by Prof. Cabell, the Chairman, before the general session.

On motion of Dr. Bell of New York, the paper was recommended for publication in the transactions.

The Chairman then read a paper on "The Bearings of Hygiene on Therapeutics," by Dr. J. R. Black, of Newark, Ohio.

On motion of the Secretary the paper was recommended for publication in the transactions.

After some discussion the section adjourned.

Upon the opening of the FINAL GENERAL SESSION at the usual hour, Friday morning, President Richardson announced the Ozone Committee appointed in accordance with a previous resolution.

The Secretary read a letter of regret from Dr. George E. Fenwick, of New York, which was ordered printed in the minutes.

Dr. J. S. Hibbard, of Indiana, offered the following resolution, which was adopted:

*Resolved*, That hereafter it shall be the duty of the Committee on Necrology to continue their reports to the death of medical men who have been members of this Association, and at the time of death were still in good fellowship or honorably separated from the Association.

Dr. A. N. Bell, of Garden City, moved the continuance of the committees appointed two years ago for the organization of State Boards of Health. The same was on motion carried.

The following communication was received from the Medical Society of the State of Pennsylvania and, after being read by the Secretary, was ordered entered on the minutes:

At the annual meeting of this body, held in Pittsburgh, May 1878, it was unanimously

*Resolved*, That the Medical Society of the State of Pennsylvania, recognizing the advantages of the metric system from its universatility, simplicity and scientific character, does recommend the use of the same to the members of the society, and urges them to familiarize themselves with it, and to advise their students to use it exclusively when they commence their medical career.

*Resolved*, That all communications made to this Society in which reference is made to weights and measures, the metric system only should be used.

*Resolved*, That the Secretary of this Society is instructed to bring this action of this society to the notice of the American Medical Association at its next meeting, and urge upon the National Association a similar action.

The preamble and resolutions were adopted.

A resolution was offered by Dr. Seguin asking the confirmation of Drs. Sims, Drysdale and Seguin as Commissioners and Delegates for the question of medical uniformity in Europe, to report next year. Adopted.

Dr. Davis next offered the following resolution which was adopted :

*Resolved*, That the section on Practical Medicine, Materia Medica, and Physiology recommend the appointment by the American Medical Association of a committee of five members, to whom shall be referred so much of the recommendations in the address of the President of that action as relates to the establishment of proper sanitaria for consumption, and the more accurate utilizing of the various mineral waters of our country with instructions to report at the next meeting of the Association.

The President announced the following delegates :

To European Medical Societies—Drs. Sims, Drysdale, Seguin, Daly, Halberstadt, Levis and W. H. Pancoast.

To the Canadian Medical Association—Drs. Brodie, Todd, E. N. Brush and W. Clarke.

The minutes of sections I., II. and IV. were received, and referred to the Publication Committee.

The following resolutions concerning certain legal relations of the Insane, offered by Dr. Foster Pratt, of Michigan, in Section V, and referred to the Association, were received and adopted :

*Resolved*, That the personal restraint of the insane is an essential element of the medical treatment of their disease, the use of which, as a therapeutical agency, may be justified by their insanity, just as the use of it as a public agency, for the prevention of injury to person or property, is justified by their dangerous conduct :

*Resolved*, That while none question the necessity for specific statutory provisions to regulate the restraint of those insane persons who are wholly or partly a public charge ; we maintain

That it is the duty of relations and friends, and it is also their natural and inherent right, whether declared or understood by statute, to restrain and to care for their sick or insane relations as a private patient at his or their expense, in his or their home, or in a legally recognized and regulated hospital ; and

That the exercise, by them, of so much restraint as is essential to their proper treatment of his disease is not a violation of his rights of personal liberty ; and

That the duty and right to exercise such remedial restraint are subject to State surveilance or legal limitations only so far as may be necessary to prevent their neglect of that duty or to punish their abuse of the right.

Dr. X. C. Scott called up the resolution of last year, creating a new Section of Opthalmology, Otology and Laryngology, to be known as Section VI, and moved its adoption. Carried.

On motion of Dr. E. Smith, of Detroit, Dr. H. Knapp, of New York, was made Chairman of the new Section, and X. C. Scott, of Ohio, was made Secretary.

Dr. Walter Kempster, of Oshkosh, Wis., Chairman of the Section on Medical Jurisprudence, Chemistry and Psychology, read a valuable paper on "The Relations of Pathology to the so-called Motor Centres," which was received with marked attention and applause. It was referred to the Committee on Publication.

The minutes of Section II. and V. were received and referred to the Committee on Publication.

The Secretary next read the annual report of the Treasurer, Richard J. Dunglison, of Philadelphia.

The report was adopted and referred to the Publication Committee.

The annual report of the Publication Committee was received and adopted.

On motion of Dr. Davis the Treasurer was instructed to pay the sum of $700 as an honorarium to the Permanent Secretary for his services.

The committee on Prize Essays reported that both prizes should be awarded to the two essays by one person. The motto, is, *Tempora mutantur et nos mutamur in illes.*"

On opening the sealed envelope the name of the successful essayist was found to be Dr. John A. Wyeth of New York City.

The Local Secretary next read the annual report of the Librarian, which was referred to the Committee on Library.

On motion af Dr. Brodie, of Detroit, the Treasurer was authorized to draw a draft of $200 for the expenses of the Library.

On motion of Dr. Davis, of Chicago, the recommendation of the Librarian adding Dr. J. M. Toner, of Washington, to the Library Committee, was adopted.

Dr. J. M. Kellar, of Arkansas, offered the following, which was laid over for one year :

*Resolved,* That in future the Committee on Nominations shall present the name of no person for appointment or election to office or position, save on the Committees on Necrology and Climatology, unless the party nominated be in attendance on the Association at the time.

Dr. J. J. Caldwell, of Maryland, offered a resolution creating a new section upon "Neurology and Electrology." Action was deferred for one year.

Dr. Maddox, of Baltimore, Md., offered another resolution, creating a section on "Diseases of the Genito-Urinary Organs, including Dermatology and Syphilis." Laid over.

On motion of Dr. Davis a resolution relating to the amendments proposed by him last year, was adopted.

Preamble and resolutions in relation to the late Prof. Joseph Henry, of the Smithsonian Institute, were offered by Dr. Toner, and adopted.

Dr. Hitchcox, of Michigan, proposed an amendment in regard to the election of officers, which was laid over until the next meeting.

Dr. Parvin, the President elect, was then introduced by President Richardson with a few complimentary remarks, and the former returned his thanks for the honor conferred in eloquent and fitting terms.

Dr. J. H. Van Deman, of Tennesee, offered a preamble and resolutions of thanks, which were unanimously adopted.

The Association at twelve o'clock, on motion adjourned *sine die.*

